# Outcome of CPAP Titration for Moderate-to-Severe OSA Under Drug-Induced Sleep Endoscopy: A Randomized Controlled Crossover Trial

**DOI:** 10.3389/fneur.2022.882465

**Published:** 2022-06-13

**Authors:** Tsai-Yu Wang, Yu-Chen Huang, Ting-Yu Lin, Yung-Lun Ni, Yu-Lun Lo

**Affiliations:** ^1^Department of Thoracic Medicine, Chang Gung Memorial Hospital Linkou Main Branch, Taoyuan, Taiwan; ^2^School of Medicine, Chang Gung University, Taoyuan, Taiwan; ^3^Department of Chest Medicine, Buddhist Tzu Chi General Hospital, Taichung Branch, Taichung, Taiwan

**Keywords:** CPAP, OSA, outcome, CPAP pressure, drug-induced sleep endoscopy (DISE)

## Abstract

**Background:**

The titration pressure of continuous positive airway pressure (CPAP) is important in patients with obstructive sleep apnea (OSA). This study aimed to understand the difference between drug-induced sleep endoscopy (DISE)-guided CPAP titration and conventional sleep center (CSC) CPAP titration in patients with OSA.

**Methods:**

In this randomized, controlled, and single-blind crossover trial, we compared the effects of 1-month CPAP treatment in patients with OSA with either DISE-guided CPAP titration or CSC CPAP titration. Twenty-four patients with OSA were recruited for the study. All patients underwent polysomnography, DISE-guided CPAP titration, and accommodation. Initially, patients were randomly assigned to receive either DISE-guided CPAP titration or CSC CPAP treatment for the first month. They were then switched to other treatments in the second month. The Epworth sleepiness scale (ESS) score was recorded at baseline, 1 and 2 months.

**Results:**

The upper limit of the pressure of DISE-guided titration and CSC CPAP titration was not significantly different (13.9 ± 0.7 vs. 13.5 ± 0.5 cm H_2_O; *P* = 0.92). The residual apnea-hypopnea index and compliance were also not significantly different between the groups. ESS score significantly improved from baseline to 1 month after CPAP treatment in both groups. Both epiglottis (anterior-posterior collapse) and tongue base collapse were significantly associated with 95% CPAP pressure (*P* = 0.031 and 0.038, respectively). After multivariate regression analyses, the epiglottis (anterior-posterior collapse) was an independent factor for 95% CPAP pressure. The incidence rate of bradycardia was 58.3%, which is a safety concern for DISE. Despite the high incidence of bradycardia, all patients with bradycardia recovered with proper management.

**Conclusion:**

Both modalities were comparable in terms of establishing the pressure settings required to treat patients. Further large-scale studies are required to confirm these results.

**Trial registration:**

https://clinicaltrials.gov/, NCT03523013.

## Introduction

Obstructive sleep apnea (OSA) is a prevalent disorder with severe cardiovascular and neurocognitive consequences that can be reduced by effective treatment (1–4). Continuous positive airway pressure ventilation (CPAP), the first line of gold standard therapy, is used to treat OSA using a pneumatic splint and increase lung volume to keep the airway open, while the airway muscles relax during sleep (5, 6). When CPAP is applied to patients with OSA during sleep, all levels of airway obstruction are anticipated to be adequately opened. However, some patients with OSA cannot tolerate CPAP because of the pressure setting. Therefore, it is important to titrate adequate pressure for patients.

Traditionally, adequate pressure has been determined by manual titration in a sleep laboratory. However, laboratory-based manual titration of CPAP is time-consuming, expensive, and labor-intensive. Therefore, Auto-CPAP titration at home under new-generation ventilator machine programs is being used to solve this dilemma. A multicenter and randomized study showed that home-based auto-CPAP titration is not inferior to traditional laboratory-based manual CPAP titration in terms of acceptance, adherence, treatment time, and functional improvements (7). Although auto-CPAP titration is non-inferior to manual CPAP titration, the former still takes weeks to achieve adequate pressure even under the modern ventilator mode (8). Drug-induced sleep endoscopy (DISE)-guided CPAP titration may be a more efficient method to solve this challenge. DISE-guided CPAP takes less than an hour to determine the adequate CPAP pressure for patients with OSA. Additionally, Civelek et al. (9) showed that the results of DISE-guided and traditional CPAP titrations were not significantly different. Furthermore, DISE has been used to simulate upper airway obstruction during sleep in terms of the level and degree of obstruction (9–15). DISE also plays an important role in accessing surgical and non-surgical treatment alternatives (16–21). However, it is still uncertain whether patients will benefit from the DISE procedure before CPAP treatment, which explores the CPAP titration level, surgical plan, and alternative treatments.

No study has compared the results of DISE-guided and doctor-guided CPAP titrations. Therefore, we conducted a randomized prospective controlled crossover trial to examine the differences between DISE-guided and doctor-guided CPAP titrations. Additionally, the relationship between CPAP pressure and location and patterns of upper airway obstruction during DISE has seldom been studied (10, 14, 15). The second primary endpoint was the identification of the independent location and pattern of upper airway collapse associated with CPAP pressure. Additionally, we also described the complication rate of the DISE procedure for safety concerns.

## Materials and Methods

This prospective, randomized, single-blind, controlled, and crossover study was performed at the sleep center of a tertiary hospital (Chang Gung Memorial Hospital) from March 2018 to December 2019. The local ethics committee of the Chang Gung Memorial Hospital approved the research protocol (NCT03523013), and each patient provided written informed consent.

### Participants

Outpatients with moderate-to-severe OSA were enrolled in the thoracic medicine department of Chang Gung Memorial Hospital. Polysomnography was performed in all patients using standard techniques. Sleep stage and arousal were scored according to the American Academy of Sleep Medicine criteria (22). All participants were naïve to CPAP therapy at the start of the study. The inclusion criteria were apnea-hypopnea index (AHI) of >15 events/h and Epworth sleepiness scale (ESS) score > 10. The exclusion criteria were central or mixed type sleep apnea, age < 20 years, severe asthma, acute asthma attack, chronic obstructive pulmonary disease (COPD), bronchiectasis, congestive heart failure Fc IV, neuromuscular disease, chronic respiratory failure, American Society of Anesthesiologists class > 3, allergy to dexmedetomidine, and history of upper airway surgery, such as uvulopalatopharyngoplasty.

### Study Design

**Figure 1:**
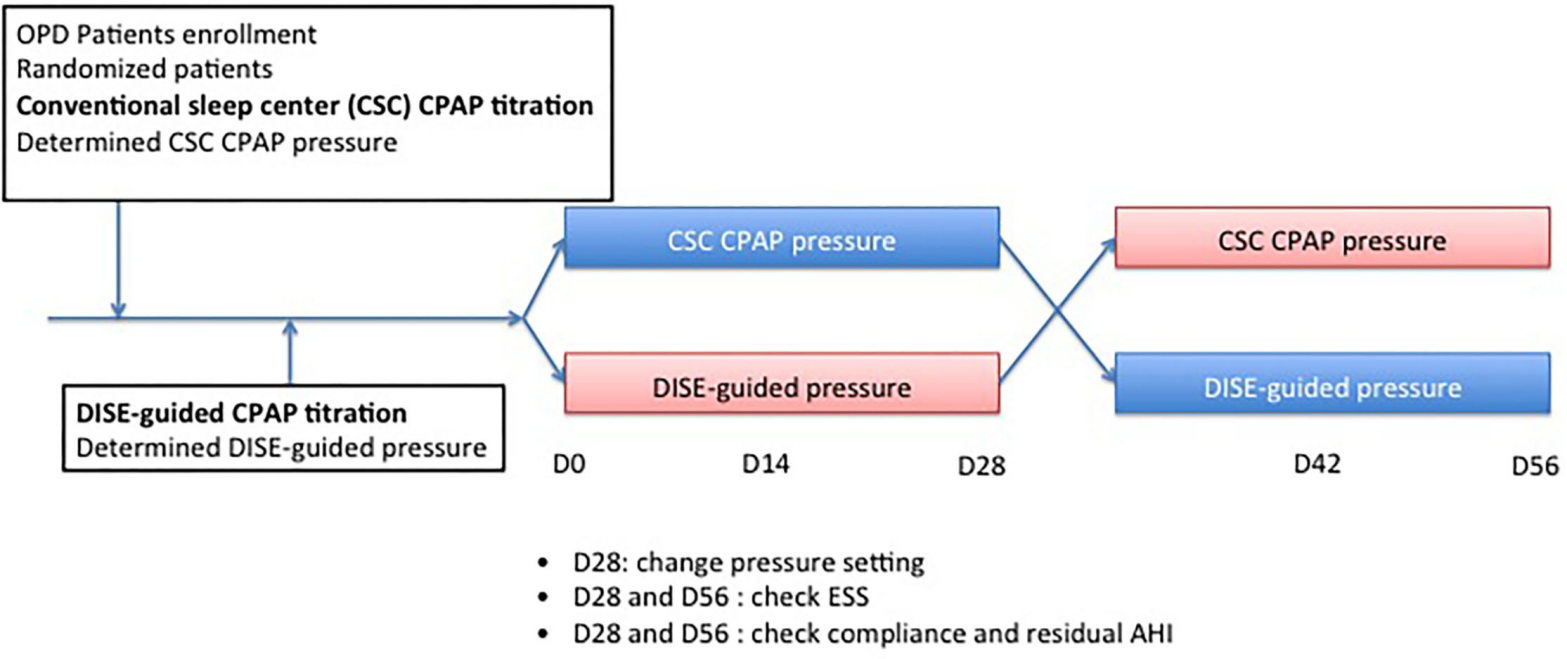
Study protocol.

This study investigated the effect of CPAP treatment with either DISE-guided CPAP titration or conventional sleep center (CSC) CPAP titration in patients with moderate-to-severe OSA. The protocol used in this study is illustrated in Figure 1. Randomization was conducted immediately after patients agreed to participate in the study. All patients underwent DISE-guided CPAP titration approximately 2 weeks later. Patients in the CSC CPAP titration group underwent auto-CPAP titration first if not contraindicated, such as those with congestive heart failure Fc IV, COPD, or neuromuscular disease; if they could not tolerate auto-CPAP titration, an overnight CPAP titration in the sleep center was arranged. Doctors determined the upper limit of the pressure auto-CPAP based on their experiences, such as body mass index (BMI), craniofacial features, and comorbidities such as allergic rhinitis or rhinosinusitis. All patients tolerated CPAP treatment in this study. In contrast, the upper limit of the pressure of the DISE-guided CPAP titration was determined during DISE. According to randomization, patients were grouped into groups A and B. In group A, patients received auto-CPAP treatment (RESMED Air Sense 10 Autoset CPAP, Bella Vista, Australia) with DISE-guided CPAP titration pressure as the upper limit of pressure for the first month. The upper limit of pressure was then modified to the CSC CPAP titration pressure for the second month. In group B, patients received CSC CPAP titration pressure for the first month. The upper limit of pressure was then shifted to the DISE-guided CPAP titration pressure for the second month. Patients were blinded to the CPAP pressure setting. Each patient was fitted with a CPAP mask and provided with instructions on CPAP operation. All patients underwent cognitive behavior therapy to maximize compliance with the CPAP therapy. All patients returned to outpatient clinics every 2 weeks to check the pressure setting. ESS score was recorded at baseline 1 month post CPAP treatment, and 2 months post CPAP treatment.

### Drug-Induced Sleep Endoscopy and CPAP Titration

Our DISE protocol has been discussed in detail previously (13). In brief, patients were placed in the supine position. One nasal cavity was anesthetized using topical xylocaine jelly. The bispectral index (BIS) (version 3.11, Aspect Medical Systems, Inc., Newton, MA, USA), electrocardiogram, blood pressure, and oxygen saturation were monitored. Drug-induced sleep was achieved using an intravenous administration of dexmedetomidine at a rate of 0.5 mcg/ (kg. h), after an initial loading dose of 1 mcg/kg, infused over 10 min. The infusion rate of dexmedetomidine was adjusted up and down by 0.2 mcg/(kg.h) to maintain the BIS level between 65 and 75. Additionally, electroencephalography (EEG), electrooculogram, and electromyography were used to monitor the sleep stages of participants during DISE. Upper airway patterns of patients were first evaluated in an awake state before dexmedetomidine injection was administered. The tip of the endoscope was held in the velopharynx during the induction of sedation to avoid further irritation. DISE was performed under a stable N2 sleep stage, with a BIS level between 65 and 75. The video-recorded examination included sequential observations of the velum, oropharynx, tongue base, and epiglottis. The CPAP pressure was increased by at least 1 cm of H_2_O at intervals of more than 2 min to eliminate obstructive apnea or hypopnea. These were determined by the simultaneous observation of flow on the CPAP mode of the bi-level positive airway pressure ventilator (Philips Respironics Trilogy 100 Ventilator Murraysville, PA, USA) and the DISE monitor screen. Apnea was defined as cessation of flow (from ventilator screen) or complete obstruction of DISE for more than 10 s. Hypopnea was defined as a 50% reduction in flow for more than 10 s, 30% reduction followed by arousal, or >3% decrease in oxygen saturation. After an adequate CPAP pressure level was achieved, sequential observations of the velum, oropharynx, tongue base, and epiglottis were repeated using the video-recorded examination.

### VOTE Classification

The VOTE classification system was used to characterize DISE findings. It focuses on specific upper airway structures that may contribute to obstruction (velum, oropharynx, tongue base, and epiglottis). At each anatomical level, the configuration of the obstruction was described as anteroposterior, lateral, or concentric. The degree of obstruction had a score for each structure: no obstruction (typically with no vibration of the involved structures); partial obstruction (typically with vibration, 25–75%); and complete obstruction (typically with total or near-total obstruction, > 75%) (11, 15). The consensus scores were determined by two investigators who were blinded to each other. They reassessed the disagreements and reached a new consensus.

### Statistical Analysis

Data are expressed as group percentages (categorical variables) or mean ± standard error (continuous variables). Categorical variables were compared using the chi-square or Fisher's exact test, where appropriate. The Wilcoxon matched signed-rank test was used for paired samples with non-normal distributions (all *P* < 0.05). The Pearson product correlation coefficient was used to examine correlations between variables and AHI or 95% CPAP pressure. Multivariate linear regression analysis was used to determine independent factors for AHI or 95% CPAP pressure. All analyses were performed using the Statistical Package for the Social Sciences software version 13.0 and Prism 5, and a value of *P* < 0.05 was considered to indicate statistical significance.

## Results

### Participant Characteristics and Polysomnography Results

The baseline patient characteristics are presented in Table 1. Twenty-four patients with moderate-to-severe OSA were recruited for this study. The patients included 22 men and two women, with an average age of 47.5 ± 2.3 years, baseline AHI of 60.4 ± 5.8 events/h, and BMI of 29.4 ± 1 kg/m^2^. Physical features such as neck circumference, Friedman tongue position (FTP), and tonsil size are listed in Supplementary Table S1. Twenty-two patients (91.7%) had high tongue positions (FTP IV).

**Table 1:** Participant characteristics.

**Characteristics**	***N* = 24**
Age, years	47.5 ± 2.3
Male, *n* (%)	22 (91.7)
Asthma, *n* (%)	2 (8.3)
DM, *n* (%)	1 (4.2)
HTN, *n* (%)	3 (12.5)
CHF, *n* (%)	0 (0)
COPD, *n* (%)	0 (0)
BMI, kg/m^2^	29.4 ± 1.0
TST, min	302.6 ± 12.4
SE, %	77.3 ± 2.7
Wake (%)	20.6 ± 2.7
N1 (%)	29.3 ± 3.8
N2 (%)	40.6 ± 3.9
N3 (%)	2.2 ± 0.68
REM (%)	10.3 ± 1.2
AHI	60.4 ± 5.8
ODI	56.4 ± 5.4
Mean O_2_	91.7 ± 1.0
Minimal O_2_	68.4 ± 3.7

*DM, diabetes mellitus; HTN, hypertension; CHF, congestive heart failure; COPD, chronic obstructive pulmonary disease; BMI, body mass index; TST, total sleep time; SE, sleep efficiency; REM, rapid eye movement; AHI, apnea-hypopnea index; ODI, oxygen desaturation index. Data are shown as mean ± standard error (SE), or n (%)*.

### VOTE Classification

The VOTE grading during DISE for both sleep and CPAP application is presented in Table 2. During sleep, 100% of the velum collapsed, with most cases involving a complete concentric collapse (18/24, 75%). Additionally, the CPAP application led to the resolution of a complete collapse in 18/18 (100%) of patients. Moreover, 16/24 (66.7%) of patients had a complete lateral collapse of the oropharynx. Considering the anteroposterior collapse of the tongue base, 16/24 (66.7%) patients had a complete collapse, while 4/24 (16.7%) patients had a partial collapse. CPAP application also led to the resolution of the complete collapse in 16/16 (100%) of patients. However, 15/24 (62.5%) of patients showed a partial collapse of the tongue base. In contrast, only 7/24 (29.2%) of patients still showed a partial collapse of the velum. For the epiglottis, both partial and complete lateral collapse of the epiglottis was observed in 33.3% of cases, which were all resolved by CPAP application. In contrast, both partial and complete anteroposterior collapse of the epiglottis was observed in 45.8% of patients; however, 25% partial collapse remained even after CPAP application.

**Table 2:** Changes in VOTE classification.

**Direction, number of patients**									
	**Anteroposterior**			**Lateral**			**Concentric**		
	**None**	**Partial**	**Complete**	**None**	**Partial**	**Complete**	**None**	**Partial**	**Complete**
**Sleep**									
Velum	20	1	3	24	0	0	4	2	18
Oropharynx				2	6	16			
Tongue base	4	4	16						
Epiglottis	13	3	8	16	4	4			
**CPAP**									
Velum	22	2	0	24	0	0	19	5	0
Oropharynx				17	7	0			
Tongue base	9	15	0						
Epiglottis	18	6	0	24	0	0			
									

*CPAP, continuous positive airway pressure*.

### Improvement in ESS Score During CPAP Treatment

The mean CSC CPAP titration pressure was 13.5 ± 0.5, whereas the mean DISE-guided CPAP titration pressure was 13.9 ± 0.7. There were no statistically significant differences between the CSC CPAP and DISE-guided CPAP titration pressure values (Table 3). After 1 month of CPAP treatment, the 95% CPAP pressure recorded from the CPAP mechanism also showed no significant differences between the two groups. Additionally, the residual AHI and compliance were not significantly different between the two groups. ESS score significantly improved from 12.5 ± 1 to 7.5 ± 0.8 in the CSC CPAP pressure group (Table 3). Additionally, ESS score significantly improved from 11.2 ± 1 to 8.3 ± 0.8 in the DISE-guided CPAP titration pressure group. The improvement in ESS score was not significantly different between the two groups.

**Table 3:** Outcomes of continuous positive airway pressure (CPAP) treatment.

	**CSC CPAP titration group**	**DISE-guided CPAP titration group**	***P-*value**
Upper limit of pressure, cm H_2_O	13.5 ± 0.5	13.9 ± 0.7	0.92
95% CPAP pressure, cm H_2_O	10.7 ± 0.5	10.1 ± 0.4	0.51
Residual AHI	1.5 ± 0.3	1.2 ± 0.2	0.52
Compliance*, %	74.2 ± 4.8	70.5 ± 5.4	0.62
ESS, baseline	12.5 ± 1.0	11.2 ± 0.9	0.40
ESS, CPAP treatment	7.5 ± 0.8	8.3 ± 0.8	0.60

CSC, conventional sleep center; DISE, drug-induced sleep endoscopy; CPAP, continuous positive airway pressure; AHI, apnea-hypopnea index; 95% CPAP pressure, 95th percentile pressure. ^*^Percentage of days of CPAP use. Data are shown as mean ± standard error (SE).

### Site of Collapse and 95% CPAP Pressure

The 95% CPAP pressure in the epiglottis-anterioposterior (epiglottis-AP) group was significantly higher than that in the oropharynx-lateral (oropharynx-L) group (12.1± 0.5 vs. 10.5± 0.5; *p* = 0.04). The 95% CPAP pressure in the epiglottis-AP group was higher than that in the velum-anterioposterior (velum-AP) group (12.1 ± 0.5 vs. 9.6 ± 1.1; *p* = 0.08) (Supplement Figure S1). In the univariate analysis, both epiglottis-AP and tongue base significantly correlated with 95% CPAP pressure (Table 4); however, they did not correlate with velum-AP, velum-lateral (velum-L), velum-concentric (velum-C), oropharynx-L, and epiglottis-lateral. Although tongue base and epiglottis-AP were chosen for analysis in the multivariate stepwise linear regression, only epiglottis-AP was an independent factor for 95% CPAP pressure.

**Table 4:** Univariate and multivariate analyses for 95% of CPAP pressure.

**Factors**	**Beta estimate**	**SE**	**95% CI**	***P*-value**
**Univariate**				
Velum-AP	0.52	0.71	−1.99–0.95	0.471
**Velum-L**				
Velum-C	0.33	0.63	−0.99–1.64	0.611
Oropharynx-L	0.75	0.74	−2.28–0.78	0.321
Tongue base	1.27	0.57	0.08–2.47	0.038
Epiglottis-AP	1.10	0.48	0.113–2.09	0.031
Epiglottis-L	0.20	0.63	−1.12–1.51	0.759
**Multivariate**				
Epiglottis-AP	1.10	0.48	0.11–2.09	0.031

*CPAP, continuous positive airway pressure; AP, anteroposterior; L, lateral; C, concentric*.

### Hemodynamic and Respiratory Complications

The incidence of bradycardia (<60 beats per min) was relatively high (14/24 patients, 58.3%) (Table 5). In contrast, the incidences of hypotension and hypoxemia were relatively low. All patients who presented with bradycardia, hypoxemia, or hypotension have recovered with proper management. None of the patients had intubation or even mortality.

**Table 5:** Proportions of bradycardia, hypoxemia, and hypotension during drug-induced sleep endoscopy (DISE).

**Characteristics**	***N* = 24**
**Bradycardia**, ***n*** **(%)**	
<60 per min	14 (58.3)
<50 per min	5 (20.8)
**Hypotension**, ***n*** **(%)**	
MAP <65 mmHg	3 (12.5)
SBP <90 mmHg	2 (8.3)
**Hypoxemia**, ***n*** **(%)**	
SpO_2_ <85%	2 (8.3)
SpO_2_ <80%	0 (0)

*DISE, drug-induced sleep endoscopy; MAP, mean arterial pressure; SBP, systolic blood pressure*.

## Discussion

The present study demonstrated that treating patients with moderate-to-severe OSA with CPAP significantly reduced subjective daytime sleepiness in both groups. There were no significant differences in CPAP pressure, residual AHI, compliance, and ESS between the two groups. Notably, epiglottis (anterior-posterior collapse) was an independent factor for 95% CPAP pressure. Hemodynamic and respiratory parameters were stable during DISE. Although some bradycardia events were noted, no cases of clinically significant bradycardia were observed in the present study. This is the first prospective, randomized controlled, single-blind, and crossover study to compare the effects of DISE-guided and CSC CPAP pressure determination on subjective daytime sleepiness in patients with moderate-to-severe OSA.

To the best of our knowledge, the current study was the first to demonstrate that there are no differences between DISE-guided CPAP and CSC CPAP in terms of titration pressure, subjective daytime sleepiness, residual AHI, and compliance. Initially, we assumed that DISE-guided CPAP titration was more efficient to titrate CPAP pressure than CSC CPAP pressure. The first step in CSC CPAP titration is auto-CPAP titration, if patients do not have any contraindications. The titration from auto-CPAP requires weeks to achieve adequate pressure even under the modern ventilator mode (8). The population of enrolled patients with moderate-to-severe OSA may be the main reason for the negative results. Sleepiness and high severity of OSA are associated with better compliance with CPAP treatment in patients (23, 24). Additionally, those with central and mixed type sleep apnea were also excluded, which may result in better compliance with auto-CPAP.

The DISE-guided CPAP titration may also offer an evaluation of the upper airway while patients are struggling with CPAP. It is important to evaluate whether the downstream flows from CPAP further push the epiglottis down into the laryngeal inlet (25–28). Previous studies have reported a wide variation in the prevalence of epiglottis collapse in patients with OSA. The prevalence ranges from 29% to 73,5% (29–31). This study revealed a 79.2% prevalence of epiglottis collapse in patients with moderate-to-severe OSA, mainly because the definition of epiglottis collapse in the current study includes both tongue-related and isolated epiglottic collapse. Additionally, 91% of patients in the present study showed tongue-related posterior displacement of the epiglottis. Similar to our results, Lin et al. (30) reported that epiglottic collapse in 94.9% of patients was tongue related. While floppy epiglottis is the etiology of isolated epiglottic collapse (32), some studies have revealed that CPAP pressure may further push the epiglottis down into the laryngeal inlet (25–27). DISE-guided CPAP titration may also be used to observe whether the epiglottis is pushed into the laryngeal inlet. However, the CPAP pressure did not further push the epiglottis down into the laryngeal inlet during DISE-guided CPAP titration in the other 9% of patients with the isolated epiglottic collapse in this study. This is another reason for the negative results of this study.

Regarding the site of collapse at atmospheric pressure for predicting CPAP pressure, 95% CPAP pressure in the epiglottis-AP group was significantly higher than that in the oropharynx-L group (12.1 ± 0.5 vs. 10.5 ± 0.5; *p* = 0.04). Additionally, only epiglottis-AP was an independent factor for 95% CPAP pressure in the multivariate stepwise linear regression, which is compatible with the findings of a previous study that found that CPAP pressure had the greatest impact on the lateral collapse of the oropharynx, and then, the velopharynx. The tongue base and epiglottis collapse were more resistant to CPAP pressure (10). However, another study revealed that velum-C and oropharynx-L were associated with CPAP titration pressure (15). This was possible because the patient populations were different. Most patients in the current study had severe OSA with a high AHI (60 events/h). Furthermore, the sample size of this study was relatively small. Further studies are warranted to confirm these results. Twenty-two patients (91.7%) had a high tongue position (FTP IV) in the current study, as well as many complete concentric collapses. A recent study identified a positive correlation between higher tongue position and complete concentric collapse, which may elucidate the findings of this study (33).

One strength of this study is the controlled sedation level according to both BIS (BIS 65–75) and EEG (N2 sleep stage). Another strength of this study is the use of dexmedetomidine, which has less influence on upper airway muscles (34) and better hemodynamic and respiratory stability during DISE. Although dose-related bradycardia due to dexmedetomidine has been reported (35), no clinically significant bradycardia was observed in this study. Controlling sedative depth using an objective tool was critical in this study. Different BIS levels are associated with different degrees of upper airway obstruction (36). Our previous study also evaluated upper airway obstruction at different sedative depths (BIS 50–60 for deep sedation vs. BIS 65–75 for light sedation) (13). We found that greater sedative depth increased upper airway obstruction. Most importantly, dexmedetomidine had less effect on the upper airway muscles and did not result in significant respiratory depression, making it suitable for DISE and bronchoscopy (34, 37–41). However, dexmedetomidine still had less effect, which may be revealed by the difference in titration pressure between auto- and DISE-guided CPAP titration. According to four randomized control trials (42–45) and three clinical series (46), the titration pressure between auto- and manual CPAP titration is generally 1–2 cm of H_2_O. Therefore, the delta titration pressure (3.8 cm of H_2_O) between auto- and DISE-guided CPAP titration is still larger than 1–2 cm of H_2_O. This may be due to the sedative agents used during the DISE. We selected dexmedetomidine rather than propofol because dexmedetomidine causes less dynamic collapse than propofol (47–50). However, the anteroposterior diameter of the retroglossal airway during high-dose dexmedetomidine infusion significantly decreased compared to that during low-dose dexmedetomidine infusion (51), which indicates that dexmedetomidine might still increase upper airway collapsibility and then increase the DISE-guided CPAP titration level.

This study was limited by its small and selected sample size, and larger studies are warranted. In addition, the study included more male patients than female patients. Therefore, the results of the present study should be interpreted with caution. Additionally, the study population mainly presented with severe OSA (AHI 60 events/h). Therefore, the results of this study may not apply to patients with mild OSA. Furthermore, this study assessed the effect of CPAP over a brief period only (1 month). Thus, further research using a larger number of individuals with a longer evaluation period is warranted.

In conclusion, the two modalities are comparable in terms of establishing the pressure settings required to treat patients. Further large-scale studies are required to confirm these results.

## Data Availability Statement

The datasets presented in this article are not readily available because of patient confidentiality and participant privacy. Requests to access the datasets should be directed to TYW (wang5531@gmail.com).

## Ethics Statement

The studies involving human participants were reviewed and approved by the local Ethics Committee of Chang Gung Memorial Hospital, research protocol NCT03523013. The patients/participants provided their written informed consent to participate in this study.

## Author Contributions

TYW and YLL designed this study. TYW, YCH, and YLL enrolled the patients and organized the database. TYW, YCH, and TYL were responsible for data analysis. TYW, YLN, and TYL performed statistical analyses. TYW contributed to the data interpretation and manuscript drafting. YLL contributed to the critical review and interpretation of data. YLL supervised the project, participated in its coordination, and helped to draft the manuscript. All authors have read and approved the final manuscript.

## Funding

This study was supported by a research grant from Chang Gung Memorial Hospital, Taiwan (CMRPG3H0381) and the Ministry of Science and Technology (MOST−108-2320-B-182-027). The funders had no role in the design of this study; the collection, analysis, and interpretation of data, or the writing of this manuscript.

## Conflict of Interest

The authors declare that the research was conducted in the absence of any commercial or financial relationships that could be construed as a potential conflict of interest.

## Publisher's Note

All claims expressed in this article are solely those of the authors and do not necessarily represent those of their affiliated organizations, or those of the publisher, the editors and the reviewers. Any product that may be evaluated in this article, or claim that may be made by its manufacturer, is not guaranteed or endorsed by the publisher.

## Abbreviations

OSA: Obstructive sleep apnea
CSC: Conventional sleep center
CPAP: Continuous positive airway pressure ventilation
DISE: Drug-induced sleep endoscopy
ESS: Epworth sleepiness scale
AHI: Apnea-hypopnea index
COPD: chronic obstructive pulmonary disease
BIS: bispectral index
EEG: Electroencephalography
BMI: body mass index
velum-AP: Velum-anterioposterior
velum-C: Velum-concentric
oropharynx-L: Oropharynx-lateral
epiglottis-AP: Epiglottis-anterioposterior.
