# Supplementary material for: Outcome of CPAP Titration for Moderate-to-Severe OSA Under Drug-Induced Sleep Endoscopy: A Randomized Controlled Crossover Trial
Source: Front Neurol. 2022 Jun 13;13:882465. doi: 10.3389/fneur.2022.882465 (PMC9234400; doi:10.3389/fneur.2022.882465)
Supplement: Supplementary file 1 [file Data_Sheet_1.PDF]

Supplementary Figure 1: Whiskers: Min to Max: 95% CPAP pressure vs. collapse group

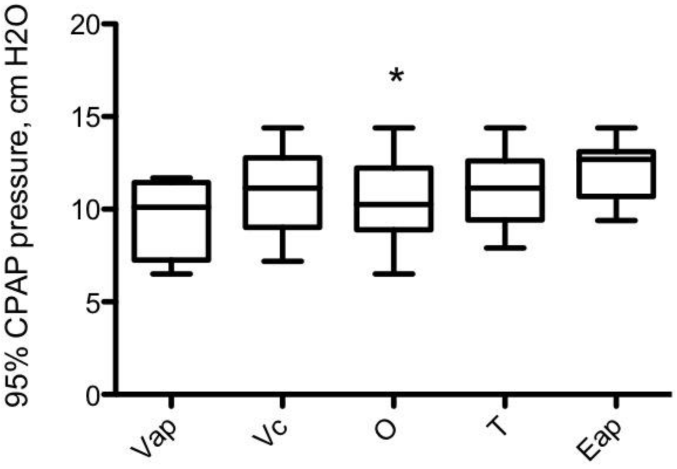

*P<0.05, Eap vs. O

Supplementary Figure 2: Whiskers: Min to Max: Residual AHI vs. collapse group

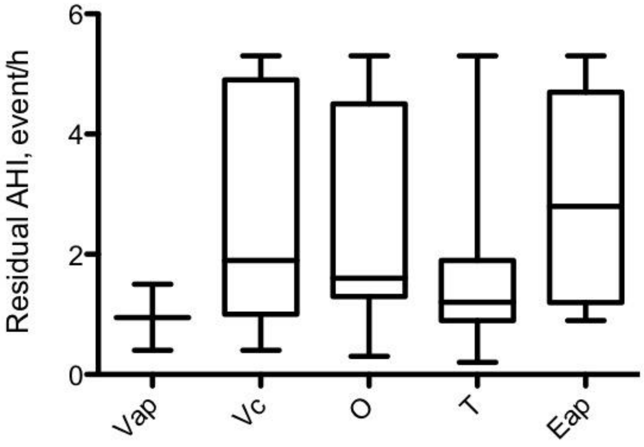

Supplementary Table 1. Participant characteristics

**Characteristics**	**N = 24**
Neck circumferences, cm	39.9 ± 3.3
**FTP**	
FTP I	0 (0%)
FTP II	0 (0%)
FTP III	2 (8.3%)
FTP IV	22 (91.7%)
**TS**	
Grade 0	3 (12.5%)
Grade 1	9 (37.5%)
Grade 2	6 (25.0%)
Grade 3	4 (16.7%)
Grade 4	2 (8.3%)

FTP, Friedman tongue position; I, uvula and tonsils completely visible; II, visible uvula and not tonsils; III, visible soft palate, but not uvula; and IV, visible only hard palate; **TS**, tonsil size; grade 0, absent; grade 1, behind tonsillar pillars; grade 2, extended to pillars; grade 3, visible beyond pillars; and grade 4, enlarged to midline.
